# Editorial: The Application of Phages Against Infectious Diseases

**DOI:** 10.3389/fcimb.2021.815984

**Published:** 2021-12-20

**Authors:** Pan Tao, Jingmin Gu, Jeremy J. Barr

**Affiliations:** ^1^ College of Veterinary Medicine, Huazhong Agricultural University, Wuhan, China; ^2^ The Cooperative Innovation Center for Sustainable Pig Production, Huazhong Agricultural University, Wuhan, China; ^3^ Hongshan Lab, Wuhan, China; ^4^ Key Laboratory of Zoonosis Research, Ministry of Education, College of Veterinary Medicine, Jilin University, Changchun, China; ^5^ School of Biological Sciences, Monash University, Clayton, VIC, Australia

**Keywords:** infectious disease, bacteriophage, phage therapy and biotechnology, pathogen detection, vaccine

Infectious diseases are a leading cause of human death. While the development of vaccines, antibiotics, and other medicines has dramatically reduced the burden of disease, multidrug-resistant bacteria and emerging infectious diseases, such as COVID-19, are reminders that continuous efforts are needed to limit these infections and their associated morbidity and mortality. Bacteriophages (phages) are viruses that specifically infect bacteria, have been utilized to combat bacterial infections since their discovery in the early 20th century. However, their clinical use was largely reduced following the discovery of antibiotics, which demonstrated greater breadth of application breadth. The emergence and rise of antibiotic resistant bacteria have forced researchers to rethink phage-based therapies as an alternative solution to tackle bacterial diseases. Significant progress has been made over the last two decades, with a number of phase I/II clinical trials having been launched or completed. Other than phage therapy, phages or their components can be used as tools for vaccine delivery and pathogen detection. Despite the tremendous advances and incredible potential, the application of phages in infectious diseases is still in its infancy. Further research and investment are required to mature phage technologies and establish phages as a viable option to combat infectious diseases. This invited Research Topic aimed to publish original articles covering such applications. This included 13 original research articles and 1 review article contributed by 115 researchers, accumulating a total of 28,228 views by the end of October, 2021.

Unlike antibiotics, phages are natural organisms that replicate within host bacteria and are subsequently released into the surrounding environment *via* lysis of the host cells. This makes them an ideal weapon to treat antibiotic-resistant bacteria. In this Research Topic, Tan et al. reported a case study of an 88-year-old patient suffering from multi-drug resistant *Acinetobacter baumannii* infection. Phage Ab_SZ3, which showed the strongest antibacterial activity among ten *A. baumannii* phages, was selected for therapeutic phage preparation. After 7 days nebulization treatment with phage Ab_SZ3 in combination with tigecycline and polymyxin E, *A. baumannii* was eliminated from the bronchoalveolar lavage fluid of the patient. Application of phages to combat multidrug-resistant bacteria in veterinary fields is also attracting attention. Mastitis is a common disease in dairy cows worldwide, which can be caused by many bacteria such as *Staphylococcus aureus*, *Escherichia coli*, and *Pseudomonas aeruginosa*. Guo et al. generated a phage cocktail containing three lytic phages, each of them demonstrating a broad host range and great bacteriolytic efficacy against *E. coli*. Using the bovine mastitis model, the authors showed that following an intramammary challenge with ampicillin-resistant *E. coli* ECD2 strain, the phage cocktail alleviated the symptoms of mastitis as efficiently as antibiotic treatment. Similarly, Wang et al. showed that intramammary injection of a lytic *P. aeruginosa* phage vB_PaeS_PAJD-1 reduced bacterial titers and repaired mammary glands in a mouse mastitis model challenged with multi-drug resistant *P. aeruginosa*.

Phages have high host specificity, which confines the application of phage therapy to sensitive bacterial strains. Therefore, phages with broad host range are highly preferred. Whittard et al. analyzed the host range of 78 lytic *S. aureus* phages using 185 *S. aureus* isolates and identified two phages, EW70 and EW71, that could infect 184 isolates. Strikingly, phage EW71 can rapidly kill *S. aureus* and reduce biofilms on polystyrene formed by methicillin-resistant *S. aureus* isolates, indicating its potential as a therapeutic candidate. Liang et al. isolated a lytic *Yersinia pestis* phage vB_YpP-YepMm, which can infect and lyse all of the three human pathogenic *Yersinia* species: *Y. pestis* (all seven strains used in the study), *Y. pseudotuberculosis* (O:1a, O:1b, and O:14), and the highly pathogenic *Y. enterocolitica* bioserotype 1B/O:8. The identification of the host receptor and the corresponding phages receptor-binding protein is important fundamental information that may provide a basis for engineering phage receptor-binding proteins and the expansion of host range. Thus, Sun et al. showed that the lytic *Vibrio cholerae* phage VP2 binds to the outer membrane protein EpsD of host cells through its tail fiber protein gp20.

The pharmacokinetics (PK) and pharmacodynamics (PD) of phage preparations *in vivo* could be key factors that determine the efficacy of phage therapy. Phages can be rapidly cleared from the blood and their residence time depends on the route of administration. Dhungana et al. evaluated the PK/PD of a lytic *Klebsiella pneumoniae* phage using oral and intraperitoneal routes in a mouse model. They found the titer of phage in plasma reached a peak at four hours after intraperitoneal inoculation, whereas the highest phage titer appeared eight hours after oral administration. However, the distribution of phages among different organs did not depend on administration route but on the presence of host bacteria in mouse. Ideally, the phage administration route should match the entry port and colonization sites of target bacteria especially for the respiratory bacteria. Wang et al. reviewed recent progresses in the aerosol delivery of phage preparations against pulmonary infections covering many technical details such as nebulization techniques, electrospray, individualized controlled inhalation, and liposome-encapsulation.

Similar to antibiotic resistance, phage therapy selects for phage-resistant bacteria. Several strategies are available to overcome this limitation, including the use of phage cocktails composed of multiple different phages, combined use of phages and antibiotics, and using phage endolysin that can directly lyse the bacterial cell wall. Song et al. showed that the lytic *Enterococcus faecalis* phage Vb_EfaM_LG1 inhibits *E. faecalis* growth *in vitro* only for four hours due to the development of phage resistance. However, the combined use of cefotaxime and phages prevented the development of phage resistance, and showed synergistic antibacterial effect against *E. faecalis*. Khan et al. reported an endolysin LysAB54 from *A. baumannii* phage p54, which showed significant bactericidal activity against several Gram-negative bacteria including *A. baumannii*, *P. aeruginosa*, *K. pneumoniae*, and *E. coli* even in the absence of outer membrane permeabilizers. Interestingly, Lu et al. found that combined use of endolysin from *S. aureus* phage P108 and vancomycin showed synergistic antibacterial effects against methicillin-resistant *S. aureus* strain XN108. Other than directly lysing the cell wall using endolysin, Blundell-Hunter et al. reported three depolymerases from *K. pneumoniae* phages that can completely remove the capsule in K-type-specific fashion. These capsules help bacteria colonize in the mammalian host and escape immune recognition and killing. Therefore, removal of the bacterial capsule might facilitate the elimination of the bacteria from the mammalian host.

Other than phage therapy, phages or its components can also be used as tools for vaccine development and pathogen detection. For example, the recombinase polymerase amplification (RPA) reaction, a widely used technology to detect nucleic acid of pathogens, was derived from the recombination-dependent DNA replication mechanism of phage T4. All three key proteins, UvsX (recombinase), UvsY (recombinase loading factor), and gp32 (single-stranded binding protein) are from phage T4. In this Research Topic, Ma et al. developed a real-time RPA technology by incorporating a fluorescence report system for the detection of *Enterocytozoon hepatopenaei*. It takes just seven minutes to detect *E. hepatopenaei* with a sensitivity of 13 gene copies per reaction. Wang et al. developed a ligation/RPA method for rapid detection of SARS-CoV2. T4 phage DNA ligase is used to ligate two DNA probes that paired with target RNA of SARS-CoV2, and ligation products are used as templates for RPA, therefore, avoiding the need for reverse transcription. The procedure can be completed within 30 min with a sensitivity of 10 viral RNA copies per reaction.

Overall, our current Research Topic covers most areas of phage therapy and partly pathogen detection. We realize that application of phages against infectious diseases is not limited to the topics discussed here. For instance, we have not received high-quality research articles regarding phage-based vaccine development due to time limit. Nevertheless, this Research Topic presents a valuable collection of articles that help mature phage technologies and establish phages as a viable option to tackle infectious diseases.

## Author Contributions

PT drafted the manuscript. JG and JB revised the manuscript. All authors contributed to the article and approved the submitted version.

## Funding

This work was supported by the Natural Science Foundation of China (Grant No. 32170094 and 31870915 to PT, Grant No. 31872505 and U19A2038 to JG), and the Shanghai Municipal Health Commission Scientific Research Project (Grant No. 20194Y0061) and the Fundamental Research Funds for the Central Universities (Program No. 2662019PY002).

## Conflict of Interest

The authors declare that the research was conducted in the absence of any commercial or financial relationships that could be construed as a potential conflict of interest.

## Publisher’s Note

All claims expressed in this article are solely those of the authors and do not necessarily represent those of their affiliated organizations, or those of the publisher, the editors and the reviewers. Any product that may be evaluated in this article, or claim that may be made by its manufacturer, is not guaranteed or endorsed by the publisher.

